# HDMap-SLRC: Semantic-Landmark Residual Correction for Monocular HD-Map Georeferencing

**DOI:** 10.3390/s26216765

**Published:** 2026-10-26

**Authors:** Eun-Joo Suk, Yo-Han Han, Gwang-Jae We, Ju-Hwan Moon

**Affiliations:** GEOSTORY Co., Ltd., Gangseo-gu, Seoul 07566, Republic of Korea; cocouki09@geostory.co.kr (E.-J.S.); snrn5008@geostory.co.kr (Y.-H.H.); gjwie@geostory.co.kr (G.-J.W.)

**Keywords:** monocular camera, HD map, georeferencing, semantic landmarks, Gaussian process, autonomous driving

## Abstract

**Highlights:**

**What are the main findings?**

- The proposed two-stage framework reduced traffic-light map-referenced error from 24.8 m to 5.9 m and pole error from 19.8 m to 11.2 m in the primary sequence. In the additional Yeouido scene, the mean of the segment-wise median horizontal trajectory errors against the RTK-GNSS/INS reference decreased from 21.2 m to 10.5 m.
- In the primary sequence, Gaussian-process correction further improved pole-to-map consistency and road lateral alignment, after geometric initialization.

**What are the implications of the main findings?**

- The evaluated monocular reconstructions can be linked to an HD-map reference, although the remaining meter-level errors do not establish suitability for lane-level autonomous driving.
- Coarse road-section prioritization for subsequent inspection and precise surveying is a potential application whose operational usefulness remains to be validated.

**Abstract:**

Low-cost monocular cameras can reconstruct road scenes in three dimensions, but the scale and local coordinate frame of the reconstruction must be linked to an absolute spatial reference. This study proposes HDMap-SLRC, an offline georeferencing framework combining HD-map centerline-based alignment with semantic-landmark residual correction. The geometric stage establishes a map-referenced trajectory, and a Gaussian-process module estimates conservative along-route corrections from sparse semantic observations. In the primary sequence, traffic-light map-referenced error decreased from 24.81 m to 5.87 m, pole error from 19.78 m to 11.21 m, and the 95th percentile (p95) of road lateral distance from 5.302 m to 2.741 m, relative to the 4-DOF baseline. Landmark metrics were interpreted as map consistency, considering the relationship between correction inputs and the evaluation procedure. Across 28 parameter configurations on two reconstruction variants of the primary sequence, the range of road lateral p95 was approximately 3.0% of its mean. In an additional Yeouido acquisition comprising four segments, 992 m, and 160 frames, the mean of the segment-wise median horizontal trajectory errors against an RTK-GNSS/INS reference decreased from 21.16 m to 10.55 m. A scale-perturbation analysis of the saved geometric stage examined how input-scale changes propagate to aligned positions. The results support the feasibility of the staged georeferencing structure under the evaluated conditions. The remaining meter-level errors do not establish suitability for lane-level autonomous driving, and generalization across weather, illumination, and urban layouts remains unvalidated.

## 1. Introduction

With the expansion of autonomous driving and road spatial-information construction, three-dimensional reconstruction of road scenes with low-cost monocular cameras has attracted attention as an economical alternative to LiDAR [1,2,3,4]. Two properties, however, prevent its direct use as spatial data: the reconstruction is recovered only up to an unknown scale, and it exists in a local coordinate frame with no absolute datum. In open-sky conditions a GNSS trajectory can supply both, but in urban canyons multipath and signal blockage make the GNSS solution unreliable precisely where dense road furniture makes mapping most valuable. Georeferencing—converting a reconstruction into measurable absolute spatial data—therefore has to rely on some other absolute reference [5], and high-definition (HD) maps are attractive candidates because they already encode road centerlines, elevations, and semantic-landmark layers such as traffic lights and poles, in an absolute frame [6,7,8].

This study addresses the correction of along-route positional deviations in a monocular road-scene reconstruction after initial alignment to an HD map, using sparse semantic-landmark observations. A single global similarity transform can determine the overall position, orientation, and scale, but cannot remove spatially varying deviations with one transform. Increasing correction flexibility can improve agreement at observed landmarks while affecting the initial road alignment. The central question is therefore how landmark-derived corrections can be propagated to the reconstructed scene while evaluating both positional improvement and preservation of geometric alignment.

HDMap-SLRC proposes a two-stage georeferencing structure that combines geometric initialization from continuous HD-map centerlines with semantic-landmark residual correction. Initialization places the reconstruction in a map-referenced road geometry, while residual correction incorporates along-route positional information from semantic observations. The correction is applied to the georeferencing of the reconstructed scene, rather than being confined to individual landmark coordinates. Based on this division of roles, the study compares correction models using object-position and road-alignment metrics together.

Recent developments in visual localization include regression-based methods that estimate camera pose from images without a prebuilt explicit 3D map [9,10], generative methods that predict the image capture location on the Earth’s surface from image features [11], and methods that use satellite imagery or OpenStreetMap as a geographic reference [12,13,14]. These methods offer alternative ways to obtain camera locations, whereas this study addresses placing a monocular scene reconstruction, including its camera positions, in a vector HD-map coordinate frame. Applying direct image-to-coordinate predictions to a reconstructed scene requires establishing the scale and transformation relating the predicted coordinates to the reconstruction coordinates. HDMap-SLRC uses continuous centerline geometry to establish the initial alignment, and sparse semantic observations to correct the remaining along-route positional deviations. This division of roles is designed to distinguish the contribution of each information source to the correction, and to propagate the resulting correction to the scene point cloud.

## 2. Related Work

Absolute localization from monocular imagery has developed in diverse directions, depending on how the scene map is represented; a recent survey systematized monocular re-localization methods by map representation, including geometric maps, point-cloud maps, and semantic maps [1]. Monocular SLAM, structure-from-motion, and monocular depth estimation form the basis of point-cloud reconstruction [2,3,4], and research on HD-map construction and semantic mapping is also advancing rapidly [8,15]. Localization and mapping in the context of autonomous driving have likewise been reviewed extensively, with the mainstream direction combining cameras, LiDAR, and prior maps [5]. Within this stream, our study focuses on the problem of aligning the reconstruction obtained from a single low-cost sensor—a monocular camera—to the HD-map coordinate frame.

Learned pose regression and geographic referencing address related but distinct questions. Reloc3r [9] estimates relative camera poses from image pairs, and combines them with a posed-image database through motion averaging. Its large-scale training supports generalization to new scenes; its authors, nevertheless, identify a degeneracy in recovering metric translation when the query and all retrieved reference cameras are collinear. SPR-Mamba [10] instead predicts poses relative to the first image in a sequence, reducing dependence on scene-specific retraining and retrieval databases. That sequence origin is a local reference, so using the result as geographic data still requires linking it to an external datum. These methods, therefore, broaden the available pose-estimation options without making explicit map-referencing requirements interchangeable.

Alternative geographic references also reduce reliance on HD maps. Zhang et al. [12] fuse visual SLAM with learned ground-to-satellite registration in a scaled pose graph, using spatial-bound and visual-odometry consistency checks to reject unreliable cross-view predictions. Their analysis identifies ambiguous longitudinal appearance and false cross-view matches as relevant failure modes. RHO [13] performs metric localization against OpenStreetMap, using panoramic imagery; its formulation uses camera calibration, gravity alignment and a coarse location prior. The authors report that its panorama-specific modules do not provide the same benefit with pinhole-only input. Compared with these approaches, HDMap-SLRC uses the existing vector centerline and semantic-object layers to transform an already reconstructed road scene. Its correction stage fits the sequence-specific anchor residuals without training a separate image-to-coordinate network, while remaining dependent on the reference map and the quality of the reconstruction and associations.

As an example of direct geographic coordinate prediction, the generative model of Dufour et al. [11] uses diffusion and flow matching conditioned on features from a frozen image encoder to estimate an image’s capture location and a distribution of possible locations on the Earth’s surface. This output is distinct from a six-degree-of-freedom camera pose or the transformation of a reconstructed point cloud. PIDLoc (2025) [14] uses ground-level RGB images, LiDAR depth, and satellite imagery, and iteratively updates position and azimuth from an initial pose. Satellite-image-based approaches should therefore also be compared in terms of their input sensors and pose-update structures. Considered alongside Reloc3r, SPR-Mamba, and RHO (2026) [13], the key distinctions concern the source of geographic reference, input sensors, estimated coordinate frame, and final output, rather than whether a method is described as single-stage or two-stage. Under the condition that a vector HD map is available, this study connects initial alignment of the reconstructed scene with residual correction from sparse observations, and computes segment-specific residuals without training a separate image-to-map-coordinate regression network.

Image-to-point-cloud registration connects observations and reference data expressed in different representations. FreeReg uses features from pretrained diffusion models and geometry from monocular depth estimation to register images to a reference point cloud [16]. In comparison, HDMap-SLRC operates on an already reconstructed road scene, and uses vector centerlines and semantic map objects as its reference. The approaches, therefore, differ in both the reference representation and the target of correction. Classical ICP [17] provides a geometric registration baseline.

Monocular imagery and semantic HD-map information have already been combined for vehicle localization. Semantic and pole-like landmarks were investigated by Sefati et al. [6]. MLVHM associates semantic–geometric image features with a vector HD map, and estimates camera pose using reprojection constraints and inter-frame motion [7]. Noizet et al. compare camera and LiDAR pole observations in a vector-map-localization filter used with GNSS and dead reckoning [18]. Wan et al. combine pole and ground-marking semantics with map reprojection and a visual–inertial pose prior [19]. Relative to these studies, the present work focuses on correction of the reconstructed scene after initial map alignment. Continuous centerline geometry establishes the initial map-referenced structure, after which sparse landmarks supply along-route residual information for correction of the reconstruction. This comparison distinguishes the estimation target and processing structure of HDMap-SLRC from related semantic-map localization methods.

GNSS–visual–inertial fusion provides another route to globally referenced localization. GVINS [20] tightly combines raw GNSS pseudorange and Doppler measurements with visual and inertial information to estimate global six-degree-of-freedom states. Its inputs include synchronized sensor observations, whereas HDMap-SLRC uses an existing monocular reconstruction and vector HD-map geometry to correct scene georeferencing. A comparison must, therefore, account for the input sensors, geographic reference, estimation target, and evaluation protocol. The present study does not include a matched quantitative comparison with GNSS–visual–inertial localization systems. This limitation is discussed in Section 5.

Recent perception and planning studies address complementary components of autonomous systems. HD-Fusion [21] integrates LiDAR and camera features through scene-level and contextual-level fusion for 3-D object detection. Wang et al. [22] improve YOLOP-based lane-line detection using feature shift aggregation in agricultural-factory environments. Yao et al. [23] use a LiDAR beam-map representation with a learned planner for local collision avoidance by unmanned surface vehicles. Although these studies do not estimate the scene-to-map transformation addressed by HDMap-SLRC, and their task-specific measures are therefore not directly comparable with the errors reported here, they can strengthen the inputs to the present framework: robust multi-sensor detection could improve landmark observations, and lane-line detection could provide denser lateral constraints for residual correction.

The conversion of road assets into spatial information provides an adjacent research context. Profumo et al. associate visually recognized road assets with GPS information for georeferencing [24]. EfMAR addresses outdoor geospatial measurement using mobile augmented reality [25], while Oh et al. use a dedicated LiDAR-observable support sign and point-cloud registration for localization correction [26]. HDMap-SLRC is distinguished by placing a monocular scene reconstruction in map coordinates using existing HD-map centerlines and semantic objects. Gaussian process regression [27] is used to incorporate sparse residual observations smoothly along the route, and sequence-alignment and motion-estimation methods [28,29,30,31] motivate alternative correction models. The contribution lies in connecting centerline initialization, semantic residual correction, and correction of the reconstructed scene within one georeferencing process, evaluated using both positional and geometric-alignment metrics.

## 3. Materials and Methods

### 3.1. Data Acquisition and Sensor Specifications

The primary video sequence was recorded using a Samsung Galaxy S26 smartphone (Samsung Electronics Co., Ltd., Suwon, Republic of Korea) in its standard video-recording mode, with automatic image stabilization enabled. Table 1 summarizes the reported acquisition and map specifications. Map-referenced horizontal positions are expressed as Easting and Northing in WGS 84/UTM Zone 52N (EPSG:32652), in meters. Automatic image stabilization is a recording setting, and does not establish that an independent geometric camera calibration was performed.

The primary sequence was recorded in daytime under clear-to-slightly-overcast skies. The reported evaluation comprises the primary acquisition and four segments from one additional Yeouido acquisition, as detailed in Section 4.3. It does not provide systematic validation under rain, snow, or night-time illumination, or across different urban layouts. Figure 1 shows the HD-map road centerlines of the test route.

### 3.2. HD-Map-Based Initial Alignment

The initial alignment places the reconstructed trajectory in the HD-map coordinate frame. In the recovered implementation, horizontal travel distance is calculated in the local XZ plane, and positive Y is treated as camera-down; this convention is not an independent gravity estimate. Let *R* index the finite road-class Y observations and *Y_a_* denote their Y coordinates. Let *Y*_cam,*i*_ denote the Y coordinate of camera position *i*. The local camera-to-road separation is the difference between the median road Y coordinate and the median camera Y coordinate. With a positive separation *y*_road_ and camera-height input *h*_cam_ in meters, the scale *s* is given in Equation (1)

(1) $$y_{\mathrm{road}}=\underset{a\in R}{\mathrm{median}}\left(Y_{a}\right)-\underset{i}{\mathrm{median}}\left(Y_{\mathrm{cam},i}\right)\;s=\frac{h_{\mathrm{cam}}}{y_{\mathrm{road}}}$$

For the primary sequence, a mounting-height input of *h*_cam_ = 1.5 m and a local camera-to-road separation of approximately 0.055 yielded a scale of approximately 27.3 m per reconstruction unit. Here, 1.5 m is the input used for scale estimation, not a statement of the measurement accuracy of the lens-center height. To first order, the relative scale perturbation equals the relative mounting-height perturbation minus the relative camera-to-road-separation perturbation. Before path clipping and semantic correction, the magnitude of the resulting traveled-distance error is approximately the traveled distance multiplied by the relative scale-error magnitude. This relationship describes error propagation at the scale-estimation stage, rather than an upper bound on final corrected positioning error. Pitch, longitudinal road grade, and systematic segmentation errors can bias the local separation when the Y axis is not aligned with the physical vertical. Section 4.4 examines this relationship numerically, using the saved geometric stage.

For frames *i* = 1, …, *n*, *s*_SfM,*i*_ is the cumulative local XZ travel distance measured from the first camera position, and *s*_SfM,end_ is the positive sequence-end distance. The dimensionless progress *u_i_* lies in [0, 1]. The prior *s*_lin_ converts this progress to metric travel distance. The centerline *C* is parameterized by cumulative horizontal distance in meters, with total length *L*_map_. The superscript (0) denotes initialization in Equation (2):

(2) $$u_{i}=\frac{s_{\mathrm{SfM},i}}{s_{\mathrm{SfM},\mathrm{end}}},s_{\mathrm{lin}}\left(u_{i}\right)=s\cdot s_{\mathrm{SfM},\mathrm{end}}\cdot u_{i}\;{q_{i}}^{\left(0\right)}=\mathrm{clip}\left(s_{\mathrm{lin}}\left(u_{i}\right),0,L_{\mathrm{map}}\right)\;{p_{\mathrm{EN},i}}^{\left(0\right)}=C_{\mathrm{EN}}\left({q_{i}}^{\left(0\right)}\right)$$

The initial position uses ${q_{i}}^{\left(0\right)}$; after semantic correction, it uses ${q_{i}}^{\mathrm{GP}}$, defined below. For either choice, write the assigned route coordinate as *q_i_*. The unit-tangent components *t*_E,*i*_ and *t*_N,*i*_ are taken from the first sample of the densified centerline whose cumulative distance is at least *q_i_*. Heading *ψ_i_* uses the East-from-North atan2 convention. The map elevation *C*_Z_ is linearly interpolated, and the camera elevation includes *h*_cam_, as summarized in Equation (3):

*p*_EN,*i*_ = *C*_EN_(*q_i_*) *ψ_i_* = atan2(*t*_E,*i*_,*t*_N,*i*_) *z*_cam,*i*_ = *C*_Z_(*q_i_*) + *h*_cam_ (3)

Because this stage assigns coordinates directly from the centerline, the Road-Z residual is interpreted as a map-consistency check metric, rather than as independent accuracy. The conventional 4-DOF alignment used for comparison does not rely on an absolute reference such as a GPS/INS trajectory; it is a two-point-correspondence 2D similarity transform (two planar translation degrees of freedom, one rotation, and one global scale) that matches the start and end points of the reconstructed camera trajectory to the corresponding start and end points of the HD-map centerline. A single similarity transform can also be applied to curved segments when the reconstructed trajectory has the appropriate shape. However, one global transform cannot remove reconstruction deviations that vary along the route. The stage-wise comparison examines how centerline-based alignment and subsequent residual correction address such spatially varying deviations.

### 3.3. Semantic-Landmark Residual-Correction Models

Semantic landmarks constrain along-route position after geometric initialization. Each observation is represented by (*ξ_j_*,*ρ_j_*,*c_j_*): normalized reconstructed progress *ξ_j_*, the anchor-implied metric route coordinate *ρ_j_*, and confidence *c_j_*. Its observed residual is *y_j_* = *ρ_j_* − *s*_lin_(*ξ_j_*), in meters. Here, *i* indexes camera frames and *j* indexes GP observations, including the two boundary observations described below. The residual function *r*(*u*) is assigned a zero-mean Gaussian-process prior with an RBF covariance kernel [27], as shown in Equation (4):

(4) $$r(u)∼\mathrm{GP}(0,k(u,u^{\prime }))\;k(u,\mathrm{u\prime})={\sigma_{f}}^{2}\exp(-\frac{{\left(u-\mathrm{u\prime}\right)}^{2}}{2l^{2}})$$

The normalized length scale is *ℓ* = 0.3, the kernel signal variance is fixed at ${\sigma_{f}}^{2}=1.0$ m^2^, and the nominal observation noise is *σ*_n_ = 5.0 m. The actual observation variances are confidence-weighted, as specified in Equation (5). The posterior residual mean is

(5) $$\hat{r}\left(u_{*}\right)={k_{*}}^{T}{\left(K|\Sigma_{n}\right)}^{-1}y\;\Sigma_{n}=\mathrm{diag}\left({\tau_{1}}^{2},\ldots ,{\tau_{M}}^{2}\right)\;{\tau_{j}}^{2}=\frac{{\sigma_{n}}^{2}}{\mathrm{clip}(\frac{c_{j}}{100},0.5,20)}$$

For *M* observations, *K*_jk_ = *k*(*ξ_j_*,*ξ_k_*), $k_{*}={\left[k(\xi_{1},u_{*}),\ldots ,k(\xi_{M},u_{*})\right]}^{T}$, and **y** contains the residuals *y_j_*. The transpose is denoted by T. The posterior is evaluated by solving a linear system, without explicitly forming the matrix inverse shown in Equation (5). In the default traffic-light extractor, *c_j_* is the detected-mask pixel count. The observations at *u* = 0 and *u* = 1 have zero residual, and *c_j_* = 10^6^. Because the confidence normalization is clipped at 20, these endpoint observations retain finite variance, and do not impose exact endpoint positions.

The posterior mean is added to the linear prior, to obtain ${\tilde{s}}_{i}$. An unweighted isotonic regression then enforces nondecreasing progress in frame order. Clipping its output to [0,*L*_map_] gives ${q_{i}}^{\mathrm{GP}}$, which is substituted for *q_i_* in Equation (3). In compact form, Equation (6) gives:

(6) $${\tilde{s}}_{i}=s_{\mathrm{lin}}\left(u_{i}\right)+\hat{r}\left(u_{i}\right)\;\hat{q}=\underset{q_{1}\leq \ldots \leq q_{n}}{\mathrm{argmin}}\sum_{i=1}^{n}{\left(q_{i}-{\tilde{s}}_{i}\right)}^{2}\;{q_{i}}^{\mathrm{GP}}=\mathrm{clip}\left({\hat{q}}_{i},0,L_{\mathrm{map}}\right)$$

This monotone regression acts on route coordinates, not on the GP kernel. With fewer than two semantic anchors or a failed linear solve, the method returns ${q_{i}}^{\left(0\right)}$. All stage-wise position and elevation evaluations use the same centerline interpolation rule.

The fixed normalized length scale *l* = 0.3 corresponds to a metric correlation length of *lss*_SfM,end_ along the uncorrected metric prior, which need not equal 0.3*L*_map_. The RBF prior correlations are approximately 0.607 and 0.135 at normalized separations of 0.3 and 0.6, respectively [27]; the length scale, therefore, describes correlation, rather than a hard cutoff. For Yeouido Segments 1–4, the corresponding metric correlation lengths are 109.60, 87.67, 57.73, and 53.50 m.

A fixed normalized length scale preserves the relative correlation structure, rather than a constant metric distance. The value of 0.3 was retained from the existing configuration, and was not reselected to minimize evaluation error. Centerline geometry defines the route, while the GP adjusts progress along it. Accordingly, geometric alignment is assessed using camera-position changes and road lateral metrics, considering observation weights, boundary observations, isotonic regression, and clipping, in addition to kernel width.

Sequence alignment provides an alternative correspondence between normalized progress profiles. Let **u** contain n normalized reconstruction-progress values and **v** contain m normalized reference-progress values. The retained implementation forms the reference by cumulatively summing median inter-frame optical-flow magnitudes and dividing by their positive total. Its first progress value is zero. With scalar pairwise cost $D_{\mathrm{ij}}={\left(u_{i}-v_{j}\right)}^{2}$, the standard Soft-DTW objective [29] is given in Equation (7)

*L*_sdtw_ = sdtw_γ_(**u**,**v**) (7)

Here, *γ* > 0 is the soft-min smoothing parameter. The cost acts on dimensionless progress sequences, rather than on a 3D trajectory and the HD-map centerline.

We refer to this semantic-landmark Gaussian-process residual-correction module as SLRC-GP. Its default settings are an observation noise of 5.0 m and a normalized length scale of 0.3. Section 4.3 compares noise values of 1, 3, and 5 m with the length scale fixed. Length-scale sensitivity is examined separately in Section 4.5. This is a sensitivity analysis on the same evaluation data, rather than independent hyperparameter validation. A further comparison model uses inter-frame image motion estimated with Farnebäck optical flow [31] for residual estimation. SLRC-GP, Soft-DTW, Farnebäck optical flow, and blended models combining these methods with SLRC-GP are compared using the same input data and evaluation procedure. However, because objects are reclustered after correction, the evaluated objects are not a fixed set of physical objects across models. Object metrics are therefore interpreted under the conditions described in Section 3.4.

### 3.4. Pole Anchors and Evaluation Metrics

Landmark evaluation in the primary sequence measures distances between estimated objects and HD-map objects. The default GP run used five correction anchors, whereas evaluation used 12 traffic-light clusters and 18 pole clusters. The corresponding cluster counts for initial-alignment evaluation were 10 and 23. These evaluation-cluster counts are distinct from the number of correction anchors. Because reclustering and nearest-neighbor association are performed after correction, Table 2 and Table 3 describe map-referenced consistency, rather than prediction errors on a fixed physical-object test set. In the anchor-composition analysis, the map IDs of poles selected for correction are excluded from evaluation. This exclusion alone does not establish independence between traffic lights and poles belonging to the same facility or between reclustered objects. The two classes can share a facility group in the map data; class separation must therefore be distinguished from physical-object separation. Road lateral distance and Road-Z residual are also internal consistency measures relative to the map used for alignment. For Yeouido, horizontal absolute trajectory error (ATE) is the Euclidean distance between estimated and reference positions in the Easting–Northing plane. The median and p95 describe the typical and upper-tail error levels, respectively, while the root mean square error (RMSE) gives greater weight to larger errors. These segment-wise summaries are reported alongside statistics for the pooled 160-frame distribution, to distinguish the aggregation methods.

### 3.5. Implementation Details

Frames are extracted from the original video at 2 fps and a resolution of 1920 × 1080. Semantic segmentation [32] and object detection [33,34] are performed on each frame, followed by clustering of traffic-light and pole objects. The median camera-to-road Y separation used for scale estimation reduces the influence of isolated outliers, but does not remove systematic mask contamination or coordinate-axis tilt. In the verified default traffic-light anchor extractor, the camera-to-detected-object observation range is limited to 40 m, while a separate 30 m matching-distance limit applies between the predicted object and its nearest map object. Repeated use of the same map object is rejected. These distance criteria serve different purposes; neither guarantees correct association at dense intersections. Evaluation measures distances between clustered landmarks and map objects separately, so evaluation distances are not restricted to the anchor-extraction thresholds. The GP computes a closed-form posterior mean conditioned on the sequence’s anchor residuals, and requires no additional neural-network retraining. Correction-module runtimes in the primary model comparison are approximately 10.7–17.7 s, distinct from the runtime of the complete image-reconstruction pipeline. The computation environment comprises an NVIDIA GeForce RTX 4060 Laptop GPU (8 GB; NVIDIA Corporation, Santa Clara, CA, USA), 32 GB of system memory, Python 3.12 (Python Software Foundation, https://www.python.org), NumPy 2.1 (NumPy Developers, https://numpy.org), SciPy 1.13 (SciPy Developers, https://scipy.org), OpenCV 4.10 (OpenCV team, https://opencv.org), and PyTorch 2.5 (PyTorch Contributors, https://pytorch.org). The primary monocular reconstruction uses a VGGT-family model.

## 4. Results

### 4.1. Comparison with the Conventional 4-DOF Alignment

Compared with conventional 4-DOF alignment, HDMap-SLRC reduced traffic-light map-referenced error from 24.81 m to 5.87 m, pole error from 19.78 m to 11.21 m, and road lateral p95 from 5.302 m to 2.741 m (Figure 2 and Table 2). The traffic-light errors for initial alignment and HDMap-SLRC in Table 2 are 5.97 m and 5.87 m, respectively. The GP further reduced the initial pole error of 17.47 m and road lateral p95 of 3.119 m to 11.21 m and 2.741 m, respectively. The contributions of the two stages are thus interpreted separately for each evaluation quantity. Road-Z residuals are 0.794 m for 4-DOF, 0.293 m for initial alignment, and 0.280 m after GP correction. Along-route correction changes the centerline position from which elevation is assigned, so Road-Z residual measures agreement with that map elevation. This comparison shows the stage-wise changes in georeferencing performance resulting from centerline-based initialization and semantic residual correction.

Figure 2 summarizes the quantitative evaluation from Table 2. In addition, Figure 3 and Figure 4 present macro- and micro-level qualitative comparisons of the reconstructed point cloud, demonstrating its spatial alignment with the HD-map centerline from plan and side perspectives.

### 4.2. Effect of Semantic-Landmark Correction and Model Comparison

In the primary sequence, SLRC-GP reduced pole map-referenced error from 17.47 m after initial alignment to 11.21 m, and road lateral p95 from 3.119 m to 2.741 m. In the model comparison using the same inputs (Figure 5 and Table 3), SLRC-GP achieved the lowest pole error. Farnebäck + SLRC-GP achieved a lower road lateral p95 of 2.730 m, compared with 2.741 m for SLRC-GP, whereas SLRC-GP ran in 10.7 s, compared with 17.7 s for the blended model. The statistical significance of the approximately 0.01 m difference was not evaluated in this single-sequence comparison. Furthermore, because object clusters can change after correction, the pole-error reduction is not interpreted as improvement on a fixed set of independent test objects. SLRC-GP is retained as the default correction module, based on the combined assessment of pole consistency, road alignment, runtime, and implementation simplicity.

### 4.3. Performance Validation of HDMap-SLRC

We evaluate the parameter sensitivity of the default correction and performance in an additional scene with a separate reference trajectory. Repeated configuration comparisons from the same acquisition are distinguished from recomputation of saved trajectories, to clarify the sample size and scope of the results. The geometric-stage analysis of input-scale changes is presented in Section 4.4.

We evaluated 24 configurations by crossing observation noise *σ*_n_ ∈ {1, 3, 5} m with the fraction of poles included as anchors {0, 0.25, 0.50, 0.75} on two reconstruction variants of the primary sequence. Four further configurations with linear-residual weight λ = 0.1, 0.2, 0.3, and 0.5 brought the total to 28 runs (Table 4). Road lateral p95 across all configurations ranged from 2.747 m to 2.831 m, with a range equal to 3.0% of the mean. This describes the between-configuration variation in road lateral p95, rather than uncertainty in all positioning errors. The lowest mean and narrowest range were observed at *σ*_n_ = 5.0 m. Pole metrics excluding the map IDs selected for correction varied with both noise and anchor composition; physical-facility independence is subject to the qualifications in Section 3.4. Road lateral p95 ranged from 2.750 m to 2.752 m across the four λ values. The length scale was fixed at *l* = 0.3. These 28 runs compare configurations of the same primary acquisition; they are not 28 independent acquisitions or randomized route-level trials. The geometric-stage analysis of input-scale changes is presented in Section 4.4.

Cross-scene applicability was investigated using an additional mobile-mapping acquisition in Yeouido, Seoul. Its map tile contains 101.2 km of centerline and 9.8 poles per km, compared with 22.8 km and 2.4 poles per km in the primary route’s map tile. Four segments were selected from one acquisition based on steady motion, reference-data quality, and map association. They comprise 992 m and 160 frames, rather than four independently acquired routes. The supplied RTK-GNSS/INS reference trajectory was snapped to the map network to designate the analyzed roads and extents, making this an evaluation under reference-assisted route selection. The 160 saved reference positions matched the coordinates of the corresponding frames in the source CSV. A horizontal uncertainty indicator was calculated as the square root of the sum of the squared Easting and Northing standard-deviation components recorded in the reference CSV. Across the 160 evaluation frames, its median was 9.40 cm and p95 was 17.36 cm; the median for the first segment was 3.35 cm.

With equal weighting of the four segment-wise medians, HDMap-init achieved 11.56 m and HDMap-SLRC achieved 10.55 m, below the saved 4-DOF summary of 21.16 m and the point-to-line ICP summaries of 21.89 m with a centerline target and 15.86 m with the full local map as the target. Mean p95 values were 40.66 m for 4-DOF, 28.36 m for initial alignment, and 24.92 m for SLRC. The segment-wise initial-alignment and SLRC values were recomputed from the saved trajectories, and are presented in Table 5. The median decreased from 16.91 m to 10.51 m in Segment 2, but increased from 9.65 m to 11.97 m in Segment 1, remained unchanged in Segment 3, and increased slightly, from 9.56 m to 9.59 m, in Segment 4. In the pooled 160-frame distribution, the median increased from 10.63 m to 10.97 m, whereas p95 decreased from 33.21 m to 27.55 m and RMSE decreased from 17.33 m to 15.35 m. In Figure 6, the bars and upper-whisker endpoints are the arithmetic means of the four segment-wise medians and p95 values, respectively.

### 4.4. Scale-Error Propagation Through the Geometric Stage

To avoid introducing additional reconstruction errors, scale perturbations of −20%, −10%, −5%, 0%, +5%, +10%, and +20% were applied, while holding the saved reconstructed trajectories and map routes for the four Yeouido segments fixed. The 0% condition exactly reproduced all 160 horizontal positions of the saved initial alignment. Geometric alignment was recomputed for 28 segment–condition combinations; image reconstruction, road-height estimation, and semantic GP fitting were not rerun. The results, therefore, characterize scale-error propagation through initial alignment. Table 6 summarizes these results.

At a −10% scale perturbation, the mean of the segment-wise medians decreased from the baseline 11.56 m to 7.86 m, whereas at +10% it increased to 17.87 m. This asymmetry reflects interaction with the existing reconstruction and route conditions; −10% was not selected as a new scale based on the evaluation reference. The maximum output displacements relative to the baseline were 34.71 m at −10% and 31.35 m at +10%. At +20%, 30 frames were clipped to the route endpoint, so the resulting limitation of output displacement is not interpreted as robustness of the method. This analysis shows how uncertainty in mounting height and camera-to-road separation propagates through scale to position.

### 4.5. GP Length-Scale Sensitivity and Residual-Prediction Validation

To investigate the effect of the length scale on residual correction, we varied it over 0.1, 0.2, 0.3, 0.5, and 0.7. For each input, the nominal observation noise of 5 m, kernel signal variance of 1 m^2^, observation-confidence processing, and auxiliary boundary observations were held fixed.

For additional validation of residual prediction, we used saved pole anchors obtained under two processing conditions for the same primary video sequence. The two conditions contained 6 and 5 anchors, respectively. Using the pole IDs in the map, we excluded one anchor at a time and predicted its residual from the remaining pole anchors, repeating this procedure for every anchor. The evaluation metric was the mean absolute error (MAE) between the predicted residuals and the saved residuals obtained through automatic map association. This comparison is a supplementary analysis of the GP posterior-mean stage before isotonic regression and clipping to the route extent, and is distinct from an evaluation of the absolute positioning accuracy of the complete pipeline. The resulting residual-prediction errors are reported in Table 7.

At a length scale of 0.3, the MAE decreased by approximately 11.0% and 15.5% under processing conditions 1 and 2, respectively, relative to the no-GP-correction condition. Among the length scales compared, the lowest MAE occurred at *l* = 0.7 (6.561 m) for processing condition 1 and at *l* = 0.3 (5.170 m) for processing condition 2. For processing condition 1, the MAE at *l* = 0.3 was 6.600 m, only 0.039 m above the minimum. Thus, *l* = 0.3 provided consistently competitive residual-prediction performance under both processing conditions.

## 5. Discussion

A key feature of HDMap-SLRC is its use of continuous road geometry and sparse semantic observations at different alignment stages. Centerline-based initialization establishes the map-referenced position and orientation of the monocular reconstruction, while semantic residual correction adjusts the along-route positional deviations remaining after initialization. In the primary sequence, initial alignment substantially improved map consistency, relative to the 4-DOF baseline. Subsequent SLRC-GP correction further reduced pole error from 17.47 m to 11.21 m and road lateral p95 from 3.119 m to 2.741 m. These results demonstrate the effectiveness of a staged approach, in which centerline geometry establishes the overall alignment structure and landmark observations refine it.

Correction-model selection should consider object positioning, road alignment, and computational cost, together. In Table 3, SLRC-GP achieved the lowest pole map-referenced error, with a correction-module runtime of 10.7 s. The Farnebäck + SLRC-GP model achieved a lower road lateral p95 of 2.730 m, compared with 2.741 m for SLRC-GP, whereas SLRC-GP was preferable in pole error and runtime. Accordingly, SLRC-GP was selected as the default correction module based on object consistency, preservation of road alignment, and computational efficiency, rather than the minimum value of a single metric.

The additional Yeouido scene was evaluated against a reference trajectory, to assess horizontal positioning error separately from map consistency. The mean of the four segment-wise medians, pooled-frame p95, and RMSE describe correction performance from different perspectives. Segment-wise statistics give equal weight to each road segment, whereas pooled-frame statistics summarize the error distribution across all evaluated positions. Reporting both distinguishes segment-dependent correction effects from changes in the overall distribution. The scale-perturbation analysis of the existing geometric stage further showed how input-scale changes propagate to along-route progress and final aligned positions. This supports the importance of a clearly defined camera-to-road separation and vertical-axis convention in height-based scale estimation.

The remaining meter-level errors limit the applications supported by these results. The 5.87 m traffic-light value is a map-consistency measure subject to the anchor composition, post-correction clustering, and association procedure described in Section 3.4; it is not an independently established absolute positioning accuracy for the complete scene. In Yeouido, the arithmetic mean of the four segment-wise median horizontal trajectory errors against the RTK-GNSS/INS reference is 10.55 m, under reference-assisted route selection. These results do not demonstrate the decimeter- or centimeter-level positioning needed for precision lane-level applications, and do not support using the present output directly for precise lane keeping or path planning. Coarse road-section prioritization for subsequent inspection and precise surveying remains a potential use. Its operational value requires application-level evaluation of positional tolerances, the target spatial unit, missed changes, and false alarms.

The empirical scope is also limited. The evaluation comprises one primary acquisition and four segments from one additional Yeouido acquisition, rather than a broad set of independently acquired routes, and complex intersections, strongly curved routes, and diverse urban layouts are not systematically covered. Performance under rain, snow, and night-time illumination has not been systematically evaluated. Under such conditions, changes in image quality and scene appearance may degrade reconstruction, road segmentation, landmark detection, and map association, reducing the number and reliability of correction anchors, even though the GP correction stage itself requires no retraining. The correction effect also varied across segments: from HDMap-init to HDMap-SLRC in Section 4.3, the pooled-frame median increased slightly from 10.63 m to 10.97 m, whereas p95 and RMSE decreased, indicating that the residual correction mainly reduced large errors, rather than improving every segment uniformly.

The experimental comparisons isolate geometric alignment and residual correction using 4-DOF alignment, point-to-line ICP, optical flow, and Soft-DTW baselines, and do not include a matched benchmark against complete visual-localization or GNSS–visual–inertial systems. Such a benchmark would require compatible sensor observations, documented calibration and synchronization, and a common protocol that distinguishes map consistency from reference-based trajectory accuracy. The reported improvements should therefore be read relative to the evaluated baselines and conditions.

## 6. Conclusions

This study proposed HDMap-SLRC, a monocular road-scene georeferencing framework combining HD-map centerline-based initialization with semantic-landmark residual correction. In the primary sequence, traffic-light map-referenced error decreased from 24.81 m to 5.87 m, pole error from 19.78 m to 11.21 m, and road lateral p95 from 5.302 m to 2.741 m, relative to conventional 4-DOF alignment. In the additional Yeouido acquisition, the mean of the four segment-wise median horizontal errors against the reference trajectory decreased from 21.16 m to 10.55 m.

The contribution lies in a structure that sequentially combines continuous centerline geometry with sparse semantic observations and applies the resulting correction to georeferencing of the reconstructed scene, beyond individual objects. Correction models were also compared, using both object-position and road-alignment metrics. Under the evaluated conditions, SLRC-GP was selected as the default module, based on pole-to-map consistency, road alignment, and computational efficiency. Correction-parameter comparisons and geometric-stage scale perturbations further characterized the response to changes in the settings.

The current empirical evaluation comprises the primary sequence and four segments from a separate Yeouido acquisition. The remaining meter-level errors do not establish suitability for lane-level autonomous driving, and the operational value of coarse road-section screening remains to be demonstrated. Future work should evaluate fixed settings on independently acquired routes under varied weather, illumination, urban layouts, and camera-mounting conditions. It should also establish test sets separated at the physical-object level, document acquisition and reference-system conditions, assess route selection without reference assistance, and include matched comparisons with advanced localization and sensor-fusion systems.

## Figures and Tables

**Figure 1:**
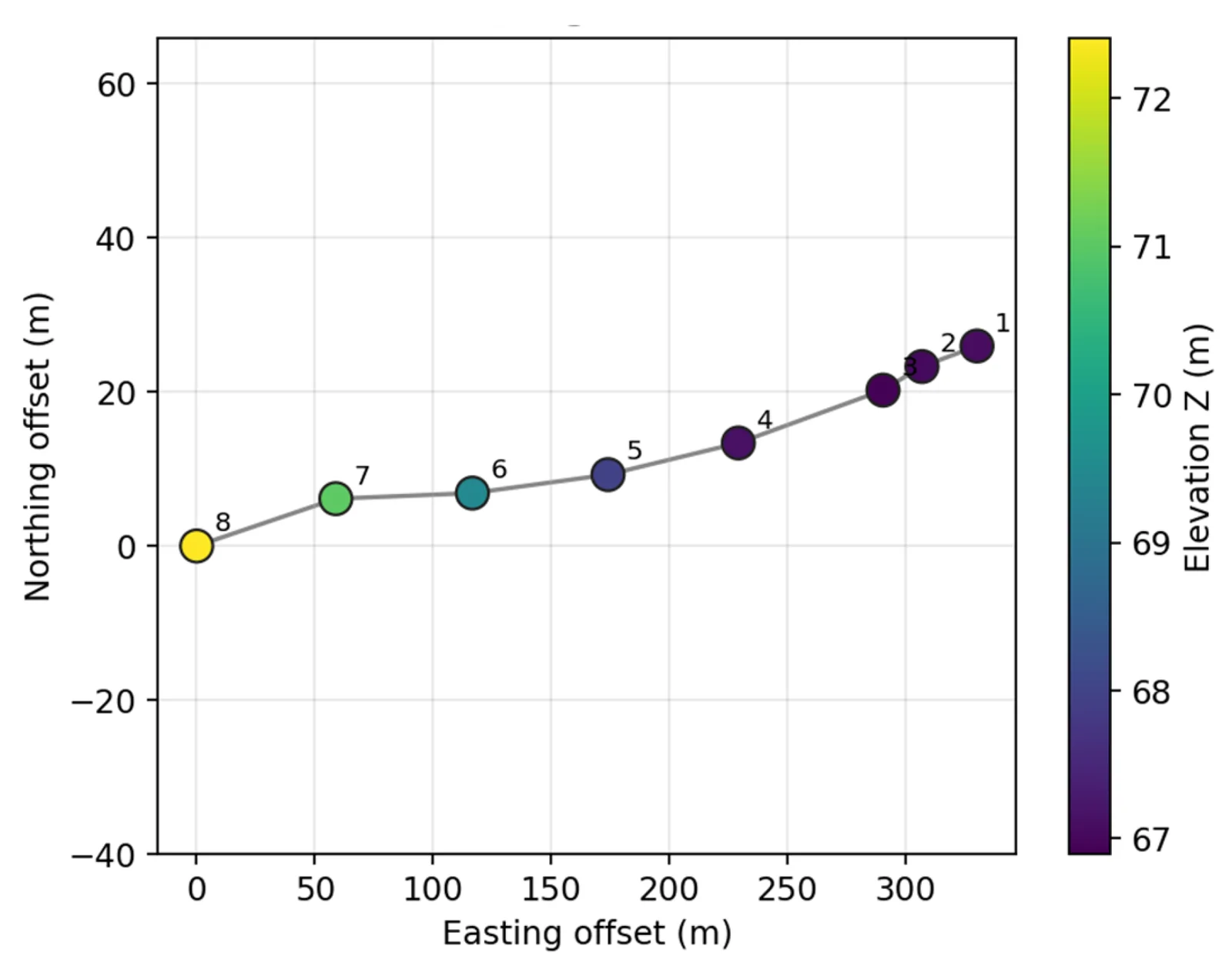
HD-map road centerlines of the test route. Color indicates elevation.

**Figure 2:**
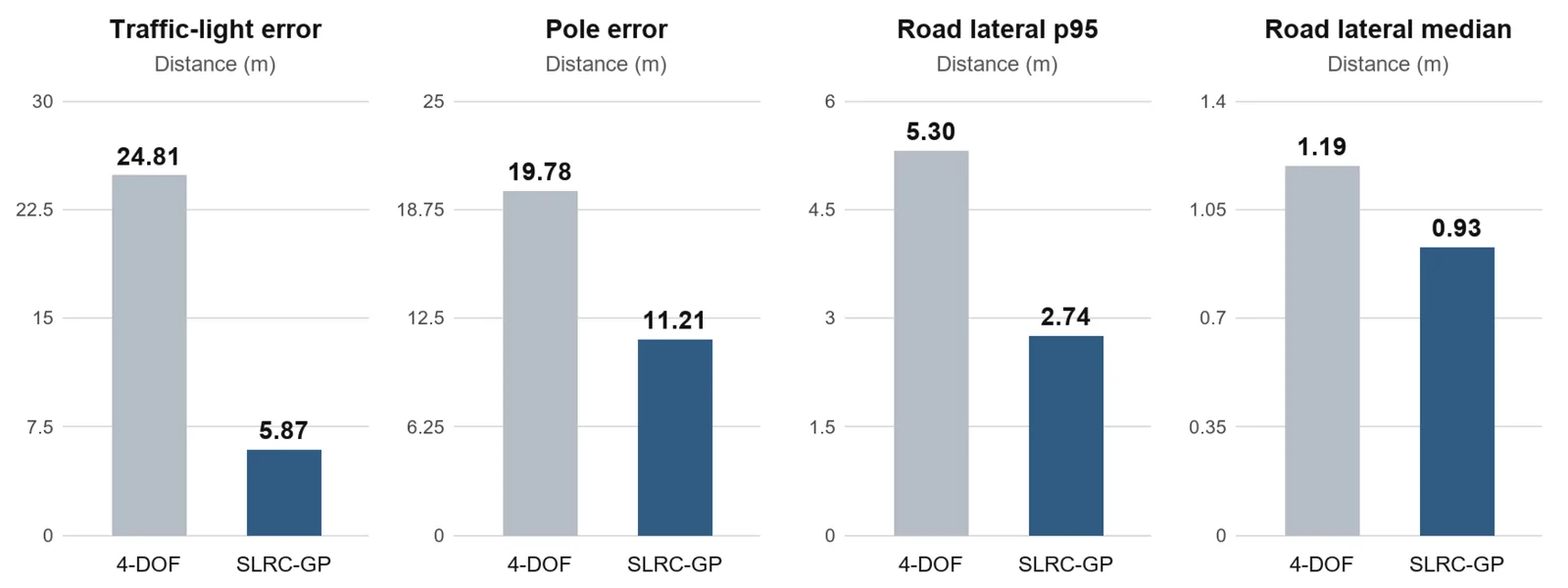
Traffic-light and pole-to-map errors and median/p95 road lateral distances for the two methods; the intermediate HDMap-init results are provided in Table 2.

**Figure 3:**
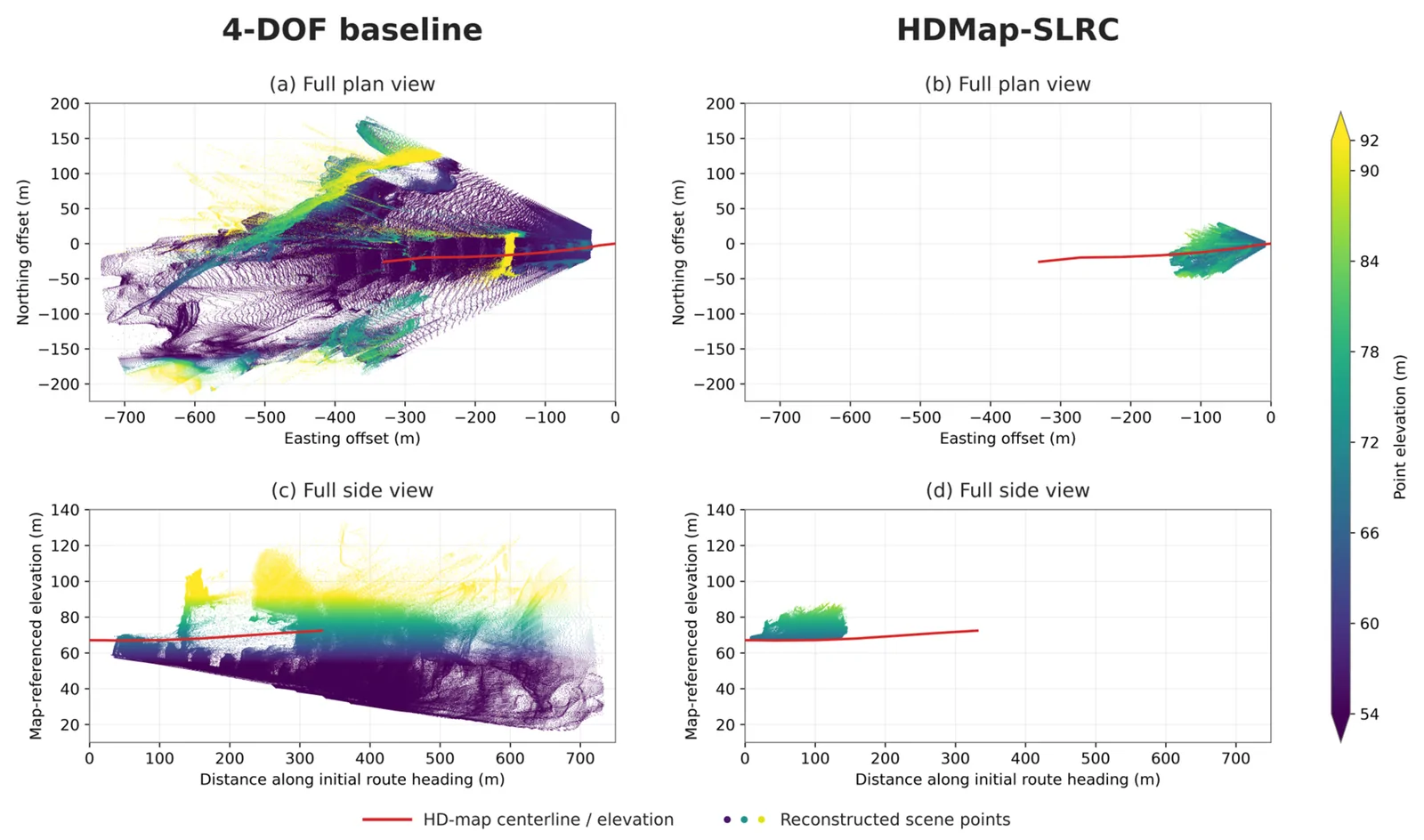
Point cloud: (**a**,**c**) 4-DOF and (**b**,**d**) HDMap-SLRC, with plan views above and side views below. Points are colored by map-referenced elevation; red lines indicate the HD-map centerline and its elevation profile.

**Figure 4:**
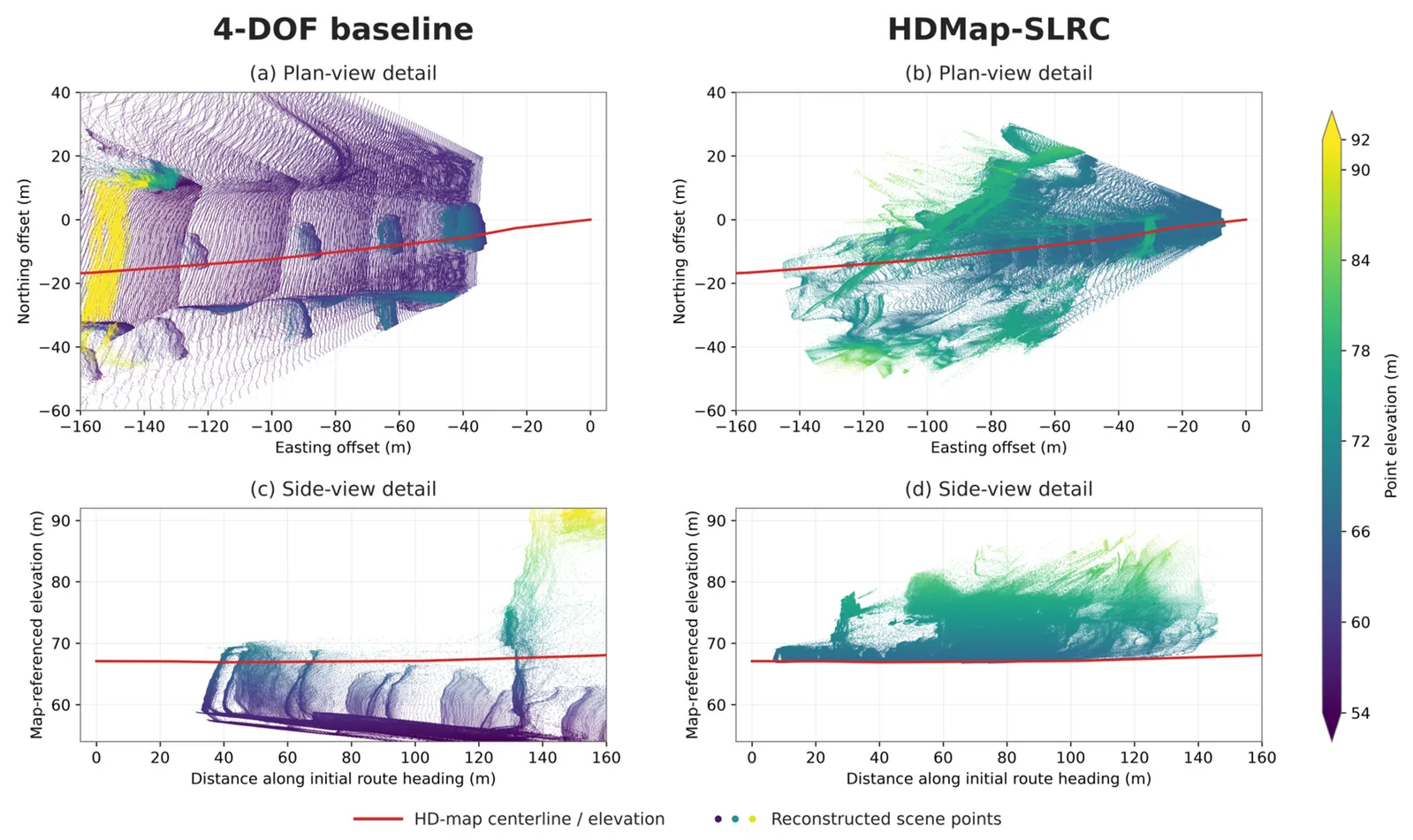
Local close-up comparison of the same point cloud shown in Figure 3. Panels (**a**,**c**) show the 4-DOF baseline, and panels (**b**,**d**) show HDMap-SLRC; panels (**a**,**b**) are plan views and panels (**c**,**d**) are side views. Corresponding panels use identical coordinate limits. As in Figure 3, point colors indicate map-referenced elevation, and red lines represent the HD-map centerline and its elevation profile.

**Figure 5:**
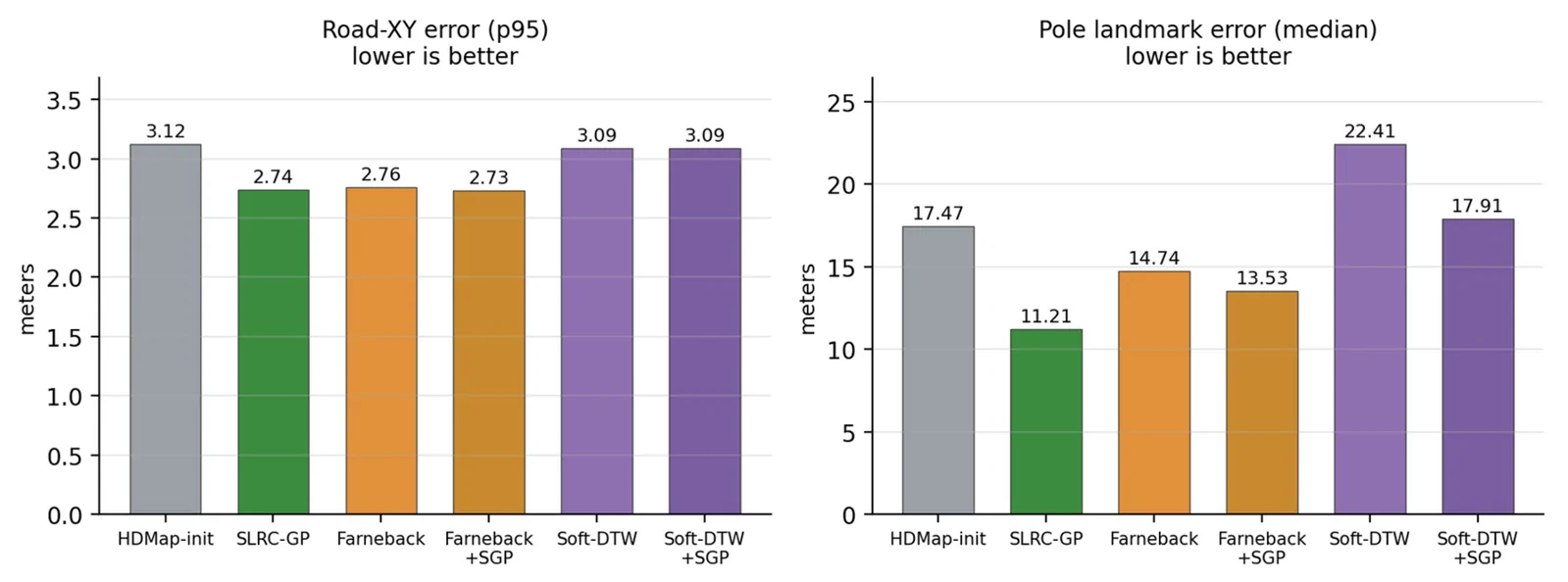
Model comparison for the road lateral error (p95) and the pole error.

**Figure 6:**
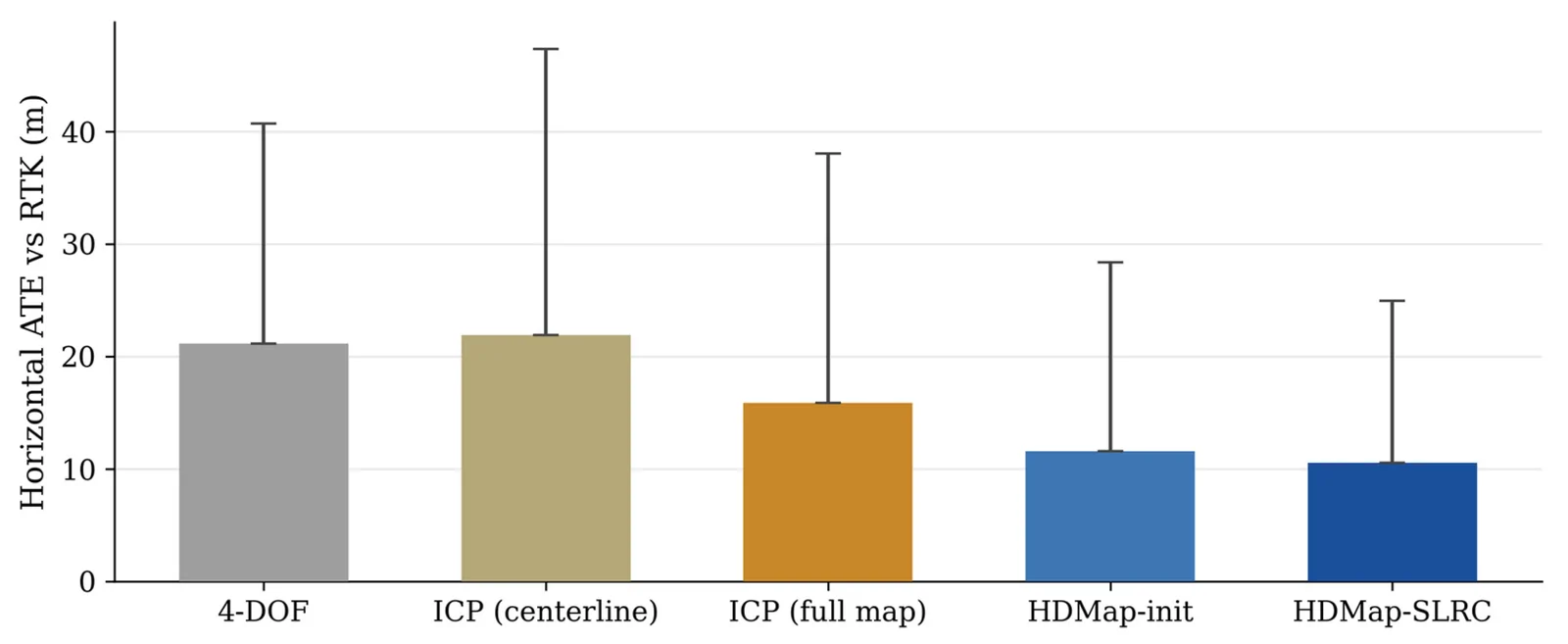
Horizontal trajectory error relative to the RTK-GNSS/INS reference for four segments from the Yeouido acquisition (992 m and 160 frames in total). Bars indicate the arithmetic mean of the segment-wise median errors, and upper-whisker endpoints indicate the arithmetic mean of the segment-wise 95th percentiles. The reference-assisted route-selection procedure is described in Section 4.3.

**Table 1:** Acquisition settings and HD-map specifications.

Item	Value
Camera model	Samsung Galaxy S26; standard video mode; automatic image stabilization enabled
Video resolution	1920 × 1080
Frame rate	Original: 30 fps; processing: 2 fps
Acquisition conditions	Daytime; clear-to-slightly-overcast skies
Camera-height input	1.5 m (scale-estimation input; measurement accuracy to be confirmed)
Estimated scale	≈27.3 m per reconstruction unit (automatically estimated road surface)
Route yaw change	≈4.2°
Road lateral evaluation samples	20,000 points (distinct from trajectory-frame count)
Road-surface points	≈1,309,000 points
Coordinate system (EPSG)	EPSG:32652 (WGS 84/UTM Zone 52N)
HD-map layers	Road centerlines, traffic lights (C1), poles (C6)
Traffic-light evaluation clusters	Initial alignment: 10/default GP: 12
Pole evaluation clusters	Initial alignment: 23/default GP: 18
GP kernel/noise	RBF; length scale 0.3; observation noise 5.0 m
Default GP correction anchors	5 (distinct from evaluation-cluster count)
Primary-trajectory evaluation samples	32

**Table 2:** Comparison of map-referenced alignment metrics for the primary sequence. Evaluation sets and the interpretation of landmark metrics follow Section 3.4.

Evaluation Metric	4-DOF	HDMap-Init	HDMap-SLRC
Traffic-light error (m)	24.81	5.97	5.87
Road lateral error, median (m)	1.188	1.005	0.925
Road lateral error, p95 (m)	5.302	3.119	2.741
Pole object error (m)	19.78	17.47	11.21
Road-Z residual (m)	0.794	0.293	0.280

**Table 3:** Comparison of machine-learning and optimization-based residual-correction models.

Model	Road Lateral p95 (m)	Pole Error (m)	Runtime (s)
HDMap-init	3.119	17.47	—
SLRC-GP	2.741	11.21	10.7
Farnebäck optical flow	2.763	14.74	15.6
Farnebäck + SLRC-GP	2.730	13.53	17.7
Soft-DTW	3.094	22.41	14.8
Soft-DTW + SLRC-GP	3.090	17.91	15.3

**Table 4:** Sensitivity to the observation noise (24 runs; two reconstruction variants × anchor compositions).

Setting	Runs	Road Lateral p95 (m)	Pole Error with Selected Map IDs Excluded (m)
*σ*_n_ = 1 m	8	2.790 (2.758–2.831)	11.06–19.99
*σ*_n_ = 3 m	8	2.768 (2.755–2.791)	12.81–20.07
*σ*_n_ = 5 m	8	2.760 (2.747–2.790)	11.19–15.13

**Table 5:** Recomputed statistics from saved trajectories for the four Yeouido segments. All errors are in meters.

Segment	Initial Median	SLRC Median	Initial p95	SLRC p95
Segment 1 (45 frames)	9.65	11.97	34.38	28.39
Segment 2 (42 frames)	16.91	10.51	33.40	25.89
Segment 3 (37 frames)	10.12	10.12	24.76	24.76
Segment 4 (36 frames)	9.56	9.59	20.90	20.64
Mean across segments	11.56	10.55	28.36	24.92

**Table 6:** Changes in geometric initial alignment under scale perturbations. Means weight the statistics of the four segments equally.

Scale Change	Mean Median (m)	Mean p95 (m)	Maximum Output Shift (m)	Endpoint-Clipped Frames
−20%	15.58	38.92	68.82	0
−10%	7.86	22.82	34.71	2
−5%	8.87	22.75	17.39	4
0%	11.56	28.36	0.00	9
+5%	14.62	33.83	16.55	14
+10%	17.87	38.77	31.35	19
+20%	25.54	50.33	57.11	30

**Table 7:** Leave-one-pole-anchor-out residual-prediction errors (MAE, m) for different GP length scales. Both processing conditions were derived from the same video sequence. For the no-GP-correction condition, the predicted residual was set to zero.

Setting	Processing Condition 1 6 Anchors	Processing Condition 2 5 Anchors
No GP correction	7.413	6.120
Length scale 0.1	7.125	6.067
Length scale 0.2	6.773	5.358
Length scale 0.3	6.600	5.170
Length scale 0.5	6.681	5.185
Length scale 0.7	6.561	5.219

## Data Availability

The data presented in this study are not publicly available, due to confidentiality agreements with a commercial partner. Derived evaluation results are available on request from the corresponding author.
